# Combining Hydrophilic Interaction Chromatography (HILIC) and Isotope Tagging for Off-Line LC-NMR Applications in Metabolite Analysis

**DOI:** 10.3390/metabo3030575

**Published:** 2013-07-18

**Authors:** Emmanuel Appiah-Amponsah, Kwadwo Owusu-Sarfo, G.A. Nagana Gowda, Tao Ye, Daniel Raftery

**Affiliations:** 1 Department of Chemistry, Purdue University, West Lafayette, IN 47907, USA; E-Mails: eappiaha@purdue.edu (E.A.-A.); kowususa@purdue.edu (K.O.-S.); ngowda@uw.edu (G.A.N.G.); yet@purdue.edu (T.Y.); 2 Northwest Metabolomics Research Center, Department of Anesthesiology and Pain Medicine, University of Washington, Seattle, WA 98109, USA; 3 Fred Hutchinson Cancer Research Center, Seattle, WA 98109, USA

**Keywords:** metabolite profiling, metabolomics, NMR, HILIC, urine, ^15^N isotope tagging

## Abstract

The complementary use of liquid chromatography (LC) and nuclear magnetic resonance (NMR) has shown high utility in a variety of fields. While the significant benefit of spectral simplification can be achieved for the analysis of complex samples, other limitations remain. For example, ^1^H LC-NMR suffers from pH dependent chemical shift variations, especially during urine analysis, owing to the high physiological variation of urine pH. Additionally, large solvent signals from the mobile phase in LC can obscure lower intensity signals and severely limit the number of metabolites detected. These limitations, along with sample dilution, hinder the ability to make reliable chemical shift assignments. Recently, stable isotopic labeling has been used to detect quantitatively specific classes of metabolites of interest in biofluids. Here we present a strategy that explores the combined use of two-dimensional hydrophilic interaction chromatography (HILIC) and isotope tagged NMR for the unambiguous identification of carboxyl containing metabolites present in human urine. The ability to separate structurally related compounds chromatographically, in off-line mode, followed by detection using ^1^H-^15^N 2D HSQC (two-dimensional heteronuclear single quantum coherence) spectroscopy, resulted in the assignment of low concentration carboxyl-containing metabolites from a library of isotope labeled compounds. The quantitative nature of this strategy is also demonstrated.

## 1. Introduction

The quantitative measurement of small-molecule metabolites present in complex biological matrices is pivotal to the field of metabolite profiling [1,2,3,4,5,6,7]. This field has garnered tremendous interest, resulting from the relatively high sensitivity of metabolite profiles to subtle stimuli, which can potentially serve as indicators of a variety of biological perturbations [8,9]. The field has shown significant potential in numerous areas, including those of medicine, toxicology, environmental and nutritional sciences, to name a few [10,11,12,13,14,15,16]. An important focus of the field is biomarker discovery in which signals from several metabolites that correlate, with a particular biological state, are combined into profiles to serve as accurate diagnostic and prognostic tools. During the process of drug development, the ability to characterize unambiguously the xenobiotic metabolites that result from the introduction of drug candidates into animal models forms the basis for advancing the drug developmental pipeline.

Nuclear magnetic resonance (NMR) spectroscopy is a ubiquitous analytical tool in metabolomics owing to its inherent quantitative, non-destructive, and reproducible nature. NMR based metabolomics involves the combination of high-resolution spectroscopic data with multivariate statistical methods, which allows for the exploration of subtle differences in sample cohorts by detecting multiple metabolites quantitatively and in parallel [17,18]. Notwithstanding the enormous benefits of NMR in the study and application of metabolomics, the issue of its low sensitivity, coupled with the spectral complexity, which normally characterizes NMR of biofluids, persistently limits the number of quantitatively detected metabolites. This limitation consequently hinders the ability to draw meaningful conclusions from the analytical data. Current advancements in the field aimed at circumventing some of these issues have included the development of specialized NMR probes such as cryogenically cooled and micro-coil probes [19,20,21,22,23]. In combination with larger magnetic fields, these probes have allowed for measurements of lower concentration chemical species to be made, owing to significant gains in signal-to-noise.

The use of chromatographic methods to simplify sample matrices by isolating metabolites of interest prior to NMR analysis has high utility for a variety of biological investigations [24,25,26,27,28,29]. This approach has also benefited from the use of sample pre-concentration techniques such as solid phase extraction (SPE) and column trapping to extend NMR detection limits significantly and thus circumvent the issue of sample dilution attributed to solvent mixing in the chromatographic step [26]. Despite these efforts, the use of LC-NMR for metabolite profiling and metabolite identification suffers from some drawbacks. The solvents used as the mobile phase for the chromatographic separation typically include water; however water invariably serves as an impediment during the ^1^H NMR measurements as it has an intensity that is 10^6^-fold higher than that of a majority of observable metabolite signals in bio-fluids. Sequences such as “WATERGATE,” “excitation sculpting”, “WET”, and “SOGGY” sequences have been employed to reduce solvent signals; however, these solvent suppression techniques have some limitations, and can attenuate analyte signals [30,31,32,33]. Although NOESY-type presaturation does not suffer from these setbacks, it works more effectively when used in the reduction of a single signal [34]. Thus, any technical innovation that can eliminate the need for one of these sequences will be extremely beneficial. One-dimensional ^1^H NMR is widely used in LC-NMR due to its high sensitivity, arising from the high isotopic abundance of ^1^H, and its large gyromagnetic ratio. However, sample pH and ion concentration has been shown to affect the chemical shift values of metabolite peaks from urine samples as well as those of solvents, which can be reduced but not completely eliminated [35]. These chemical shift variations may potentially lead to errors in peak assignments and challenges in solvent suppression.

The emergence of targeted metabolite profiling is potentially promising in addressing some of these challenges [36]. NMR-based targeted metabolite profiling has been improved by the use of a chemoselective tag (usually an isotope label) that can specifically target certain classes of metabolites, including amino acids, lipids, carboxylic acids, and metabolites with active hydrogen moieties. The common isotopes that are used for this purpose include, but are not limited to, ^31^P, ^13^C, ^19^F, and ^15^N [37,38,39,40]. For example, the use of ^15^N-ethanolamine to “tag” metabolites with carboxyl groups selectively was demonstrated recently [40]. This approach allowed the detection of well over 100 metabolites from a single class with limits of detection of a few μM in human urine and serum. The quantitative and reproducible nature of the derivatization approach provides a basis for routine investigations. A drawback, however, is that while many signals are readily detected in the 2D HSQC (two-dimensional heteronuclear single quantum coherence) NMR spectrum of urine, the identity of many of the chemical species remains unknown. This is due to the fact that new molecules that are synthesized have unique chemical shifts. Notwithstanding these limitations, the derivatization approach is potentially useful for the elimination of the problems of pH dependent chemical shift variations and solvent signal overlap issues that are more prevalent in 1D ^1^H-NMR, as the detection of the derivatized compounds utilizes heteronuclear ^1^H-^15^N 2D HSQC. Any remaining chemical shift variations are less problematic due to the improved spectral resolution and reduced overlap of these multi-dimensional experiments. Additionally, solvent signals have minimal effects on the detection of signals in the heteronuclear ^1^H-^15^N 2D HSQC experiment, which makes this approach advantageous for the detection and subsequent identification of lower concentration metabolites [41].

Hydrophilic interaction chromatography (HILIC) has been featured in a number of metabolite profiling applications due to its excellent retention of polar metabolites [42,43]. In this work, we present a reproducible two-dimensional HILIC separation approach for resolving and detecting several similarly polar and ^15^N ethanolamine tagged metabolites by ^1^H-^15^N 2D HSQC NMR. This approach facilitated the unambiguous assignment of low concentration metabolites that were not previously identified. The use of HILIC-NMR for the separation and identification of chemically derivatized metabolites in human urine encompasses the benefits of traditional LC-NMR of bio-fluids (such as reduced spectra complexity), while reducing the effects of pH dependent chemical shift variations that are commonly associated with 1D ^1^H-NMR detection and the concomitant solvent masking of lower intensity signals. Additionally, the derivatized compounds appear chemically stable on the chromatographic column, thus making this approach well suited for metabolic profiling applications. The ability to quantify ^15^N ethanolamine derivatized metabolites in human urine is also demonstrated.

## 2. Experimental Section

### 2.1. Reagents and Biological Samples

Deuterium oxide (D_2_O, 99.9%) and ^15^N-ethanolamine were obtained from Cambridge isotope Laboratories Inc (Andover, MA). HPLC-grade acetonitrile (ACN, 99.8%) and ammonium hydroxide (28%–30% NH_3_) were purchased from Mallinckrodt Baker, Inc. (Phillipsburg, NJ, USA), and sodium azide was obtained from Fischer Scientific (Pittsburgh, PA, USA). Carboxyl containing metabolites (Table 1), 3-(trimethylsilyl) propionic acid-2,2,3,3-d_4_ (TSP), and sodium acetate were obtained from Sigma Aldrich (St Louis, MO, USA). All reagents were used without additional purification. Deionized water was obtained from an EASY pure II UV water purification system (Barnstead International, Dubuque, IA, USA). Human urine samples were obtained from a healthy volunteer in accordance with a protocol approved by the Institutional Review Board at Purdue University. Sodium azide (0.1%, w/v) was added to freshly collected urine to prevent bacterial growth. Urine was purified by centrifugation using Centriprep filters with a nominal molecular weight limit of 10,000 (cat, No. 4321, Millipore, Bedford, MA, USA), aliquoted, and frozen at −80 °C until used. 

**Table 1:** 15N and ^1^H chemical shift values for the identified metabolites in human urine by combining 2D HILIC-LC and ^1^H-^15^N 2D HSQC NMR.

Label	Metabolite	^1^H (ppm)	^15^N (ppm)
1	Hippuric acid	8.12	114.94
2	Glutamic acid	8.20	115.67
3	Suberic acid	8.01	119.34
4	Cis-aconitic acid	8.18	120.41
5	4-Hydroxylphenyl acetic acid	8.10	120.84
6	3- Hydroxybutyric acid	8.04	122.02
7	Citric acid	8.03	122.77
8	Adipic acid	8.01	119.34
9	2- Hydroxyphenyl acetic acid	7.91	119.68
10	Citraconic acid	8.02	121.07
11	Phenylacetic acid	8.10	120.92
12	L- Tartaric acid	8.35	116.21
13	β-Alanine	8.10	120.07

### 2.2. ^15^N-Ethanolamine Tagging Procedure

**Figure 1:**
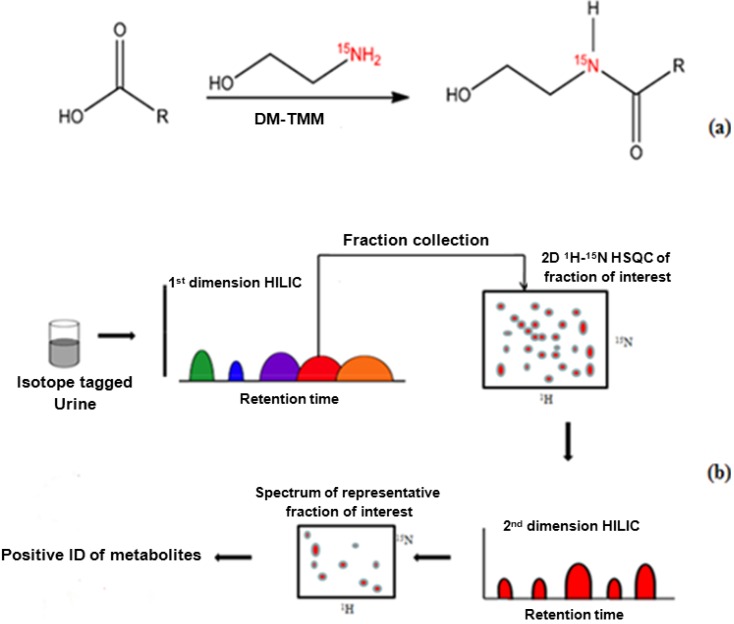
(**a**) ^15^N-ethanolamine derivatization procedure used for isotope tagging of urine metabolites [40]; (**b)** Schematic diagram of the experimental procedure for the two-dimensional LC-2D NMR method.

^15^N-ethanolamine (11 µL, 183 µmol) was added to 1.5 mL of a urine sample that had been concentrated by a factor of nine in a dry glass vial, and the pH of the mixture was adjusted to 7.0 with 1 M HCl. DMT-MM 4-[4,6-dimethoxy-1.3.5-triazin-2-yl)-4-methylmorpholinium chloride] (63 mg) was added to initiate the reaction following the procedure described by Ye *et. al*. [40,44,45] The mixture was continuously stirred at room temperature for 4 h to complete the isotope tagging reaction. In order to maintain the ^15^N amide protonation for ^1^H NMR detection, the pH was adjusted to 5.0 by adding 1 M HCl or 1 M NaOH. The reaction is summarized in Figure 1a. Spiking experiments were performed to confirm the identified peaks using 1.5 mL urine samples that were split in thirds (500 µL each). Two portions were spiked with mixtures of stock solutions of standard compounds (100 µL) and one portion (unspiked) was used as the control.

### 2.3. Two Dimensional HILIC Separation and Fraction Collection

The HPLC system was comprised of an LC-10AS Pump, SPD- 10A UV-Vis detector, SCL-10A system controller (Shimadzu Corporation, Kyoto, Japan), and a 6-port injection valve (Rheodyne, CA, USA). Fused silica tubes, 125 µm ID, and stainless steel fittings were used as the transfer lines and connectors, respectively (Upchurch Scientific, WA, USA). The HPLC system was operated using Shimadzu EZStart 7.2 software. Both dimensions of analytical separation were performed on 250 mm × 4.6 mm TSKgel Amide-80 normal phase/HILIC column (Tosoh Bioscience, Montgomery, PA, USA). A gradient elution was utilized for the first dimension separation beginning with an initial solvent composition A (90% ACN, 10% 100 mM NH_4_OH in H_2_O) and changing to composition B (60% ACN, 40% 100 mM NH_4_OH in H_2_O) over 12 min. A second gradient to composition C (40% ACN, 60% 100 mM NH_4_OH in H_2_O) was achieved in 27 min. A steep linear ramp to composition D (90% ACN, 40% 100 mM NH_4_OH in H_2_O) was achieved in 6 min, this solvent mixture was maintained for 5 min. The flow rate was 0.9 mL/min and a 100 µL injection volume was used. The separation was monitored at 254 nm with the UV-Vis detector. Fractions were collected over 6 min intervals using a Gilson FC-203B fraction collector (Gilson, Middleton, WI, USA). Fractions were collected 10 times (using 100 µL injections) and dried with an Eppendorf Vacufuge plus concentrator (Eppendorf, Westbury, NY, USA).

Following the identification of the fraction of interest by NMR (see below for description), another dimension of separation was necessary. The second protocol utilized was the following: An initial solvent composition of (85% ACN, 15% sodium acetate buffer, pH 4.0) was changed to a final solvent composition of (78% ACN, 22% sodium acetate buffer, pH 4.0) over 5 min. A second gradient to (75% ACN, 25% sodium acetate buffer, pH 4.0) was performed over a time of 7 min. This solvent composition was maintained for 12 min, followed by a final gradient in order to achieve a solvent composition of (69% ACN, 31% sodium acetate buffer, pH 4.0) in 2 min. A steep linear ramp to (90% ACN, 10% sodium acetate buffer, pH 4.0) was achieved in 1 min to serve as the wash step. The fractions for this dimension were collected over 2 min intervals resulting in a total of 11 fractions.

### 2.4. NMR Spectroscopy

The samples for NMR analysis consisted of either intact derivatized urine (500 µL) or dried chromatographic fractions reconstituted in 500 µL H_2_O. The samples were mixed with 30 µL D_2_O containing TSP (0.5 wt %/vol) for locking and chemical shift referencing, and placed in 5 mm NMR tubes. All NMR experiments were performed on a Bruker Avance DRX 500 MHz spectrometer, equipped with a 5 mm TXI triple resonance Z-gradient cryoprobe. All spectra were acquired at room temperature. One-dimensional ^1^H-NMR spectra were obtained using a 1D NOESY pulse sequence incorporating presaturation for water suppression during the relaxation delay and mixing time of 2 s and 100 ms, respectively, with a presaturation power of 50 dB in order to achieve complete water peak saturation. Thirty-two free induction decays were averaged for each spectrum. 

Sensitivity enhanced ^1^H-^15^N 2D HSQC experiments utilized an INEPT transfer delay of 5.5 ms corresponding to a ^1^J_NH_ of 90 Hz. Phase sensitive data were obtained using acquisition in echo-antiecho mode. Spectral widths of approximately 6 kHz for the ^1^H dimension and 2 kHz for ^15^N were used at 500 MHz. A total of 256 free induction decays of 2048 data points were collected in the indirect (t_1_) dimension with 32 transients per increment. ^15^N decoupling during the direct detection dimension (t_2_) was achieved with the GARP (globally optimized alternating-phase rectangular pulses) sequence. The resulting 2D data were zero-filled to 1024 points in the t_1_ dimension after forward linear prediction to 512 points. A 45°-shifted sine-bell window function was then applied to both dimensions before Fourier transformation. NMR data were processed using Bruker Topspin 2.0 software on a Redhat Linux platform. The entire experimental procedure is summarized in Figure 1b.

## 3. Results and Discussion

**Figure 2:**
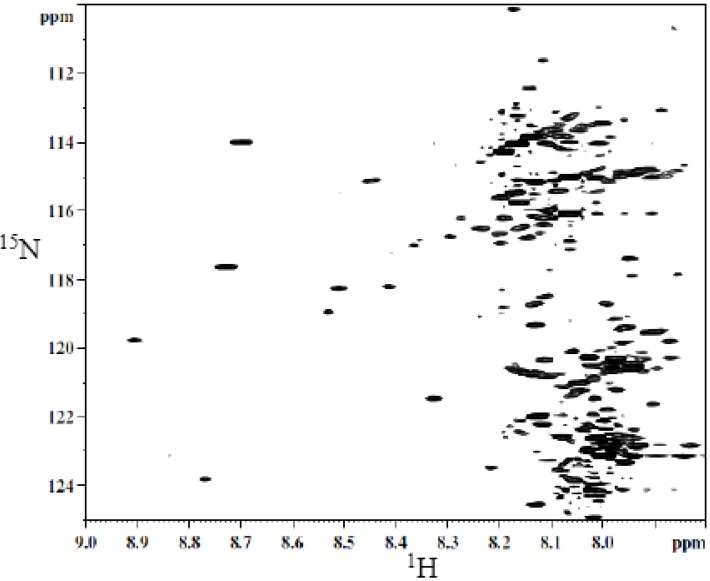
Typical 2D ^1^H-^15^N HSQC spectrum of human urine after isotope tagging using ^15^N-ethanolamine.

A typical 2D HSQC ^1^H-^15^N spectrum of derivatized whole urine is shown in Figure 2. The derivatization shows a large number of well-resolved peaks, a number of which have been identified [40]. However, the identity for a majority of the peaks is unknown. Hydrophilic interaction chromatography (HILIC) was used to fractionate the urine sample to simplify the spectrum and facilitate the unambiguous assignment of isotope labeled metabolite signals.

**Figure 3:**
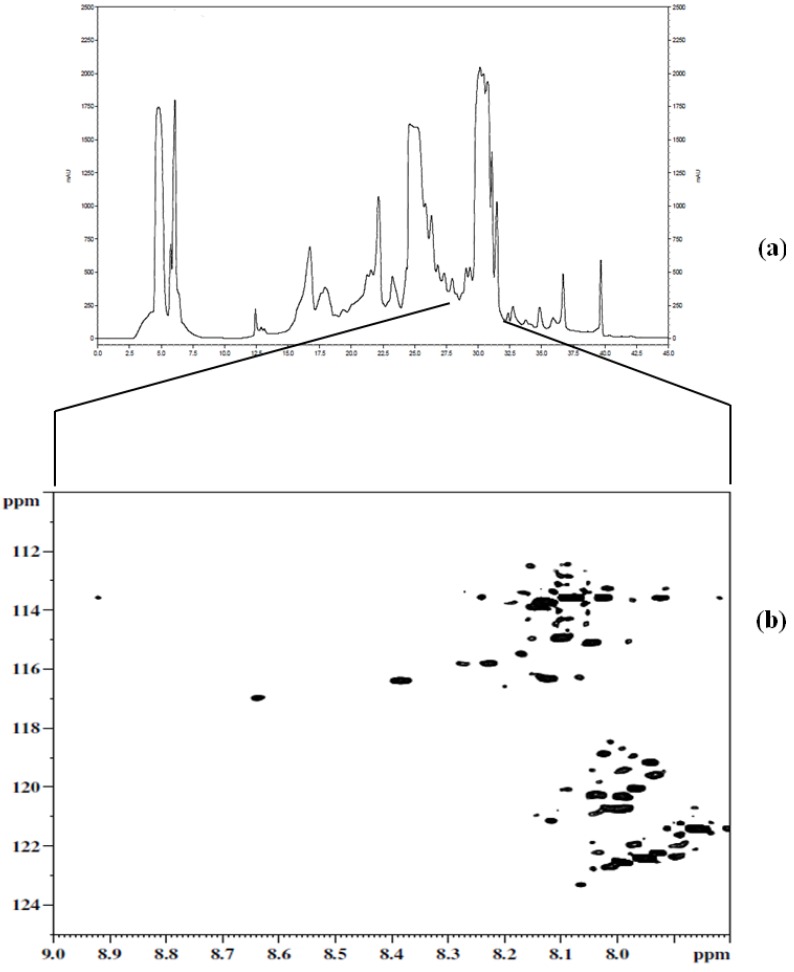
(**a**) Chromatogram of the first dimension separation of ^15^N-ethanolamine derivatized urine; (**b**) 2D ^1^H-^15^N HSQC spectrum of region of interest (*i.e.*, 27 min to about 32 min) from the first dimension separation.

Figure 3a shows a chromatogram of ^15^N-ethanolamine derivatized urine separated on a TSKgel Amide-18 HILIC column. This chromatogram shows a separation over a run time of approximately 45 min with the last peak eluting at 40 min. In order to establish the region of interest (i.e., the location of tagged compounds) fractions were collected over 6 min time intervals and spanning the whole chromatogram. The fractions were collected multiple times (10 times) using 100 µL injections, then concentrated and reconstituted in a mixture of H_2_O and D_2_O, and finally pH adjusted to 5.0 in order to maintain the protonation of the ethanolamine tagged compounds for NMR analysis. Following the NMR analysis of all fractions, it was determined that approximately 90% of the detected tagged compounds appeared in the region of the chromatogram that extended from 27 min to about 32 min, as can be seen in Figure 3b. The results from this step suggested the need for another separation procedure as many of the signals of interest co-eluted. Other components of the sample matrix largely appeared in the other fractions, which was a positive development in that it resulted in only one fraction that consisted of the analytes of interest. Thus, the tedium of a second cycle of method development was minimized.

**Figure 4:**
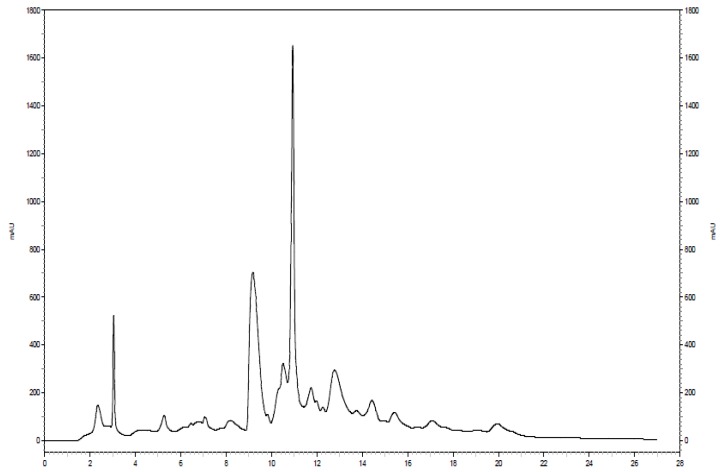
Chromatogram of the second dimension separation of a fraction of interest from the ^15^N-ethanolamine derivatized urine sample.

**Figure 5:**
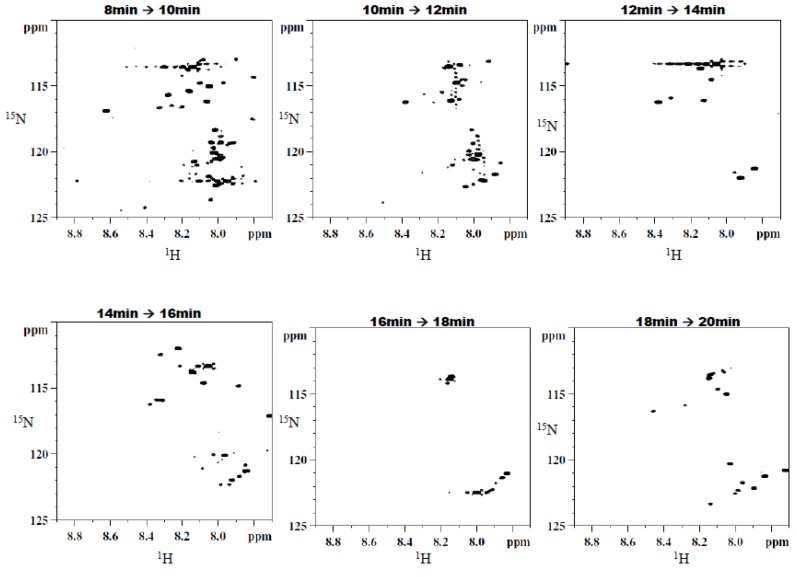
Typical 2D ^1^H-^15^N HSQC spectra of fractions resulting from the second dimension separation showing a significant simplification of the spectral data.

Further separation of the fraction of interest can be seen in Figure 4. The same analytical column was used for both separations; however, the solvent system was modified slightly in the second separation. The 100 mM NH_4_OH in H_2_O was replaced with a sodium acetate buffer at pH 4.0 to serve as the aqueous solvent. Additionally, a higher flow rate (1.9 mL/min) was utilized, resulting in a chromatogram with a total run time of approximately 22 min. Eleven two-minute fractions were collected, corresponding to the entire span of the chromatogram, and 100 µL injection volumes were used for each of 10 runs. The last six of the 11 fractions showed the presence of ^15^N-ethanolamine derivatized metabolites. The six simplified 2D ^1^H-^15^N HSQC spectra that correspond to these fractions can be seen in Figure 5. Sufficient signal was obtained for the chromatographic fractions in a shorter time as a result of the negligible effects of solvent overlap.

**Figure S1:**
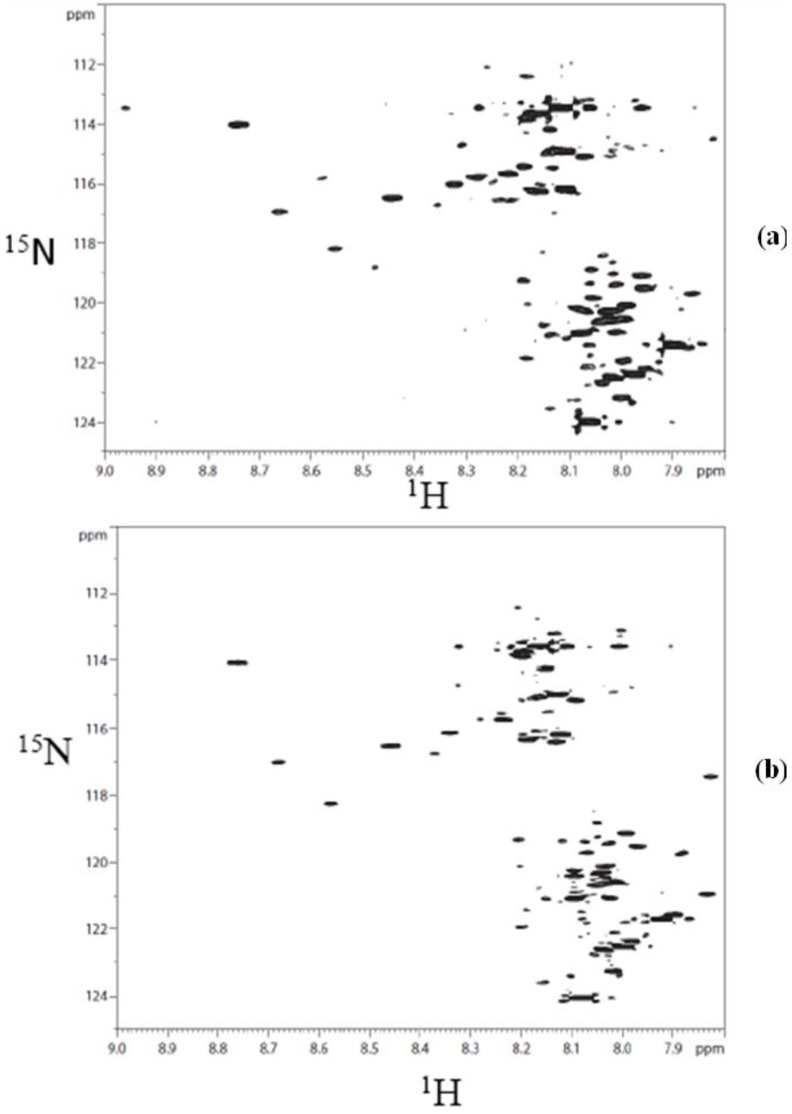
(**a**) 2D ^1^H-^15^N HSQC spectrum of derivatized urine collected after passing through TSK gel amide80 column; (**b**) 2D ^1^H-^15^N HSQC spectrum of urine taken directly after derivatization.

The choice of the TSKgel Amide-80 HILIC column for this study was based on the inherent chemical stability it offers for the separation of derivatized components in a complex biological matrix such as urine. This is illustrated in Supplementary Figure S1, which shows ^1^H-^15^N HSQC spectra of a derivatized urine, injected and collected after a chromatographic run and that of the same urine sample obtained before passing through the column. The similarity of the two spectra is indicative of the chemical stability of the analytical column used for separating the derivatized metabolites.

### 3.1. Metabolite Assignment and Quantitation by Spiking Experiments

Metabolite assignment was facilitated by the use of the simplified 2D HSQC spectra using the chromatographic fractions along with our current library of chemical shift values from ^15^N-ethanolamine derivatized standards of carboxyl containing metabolites [40]. By overlapping data points from the 2D HSQC spectra of the chromatographic fractions, and the known chemical shift values of standards from the library of compounds, 13 metabolites were first tentatively assigned. This was possible because of the lower susceptibility of 2D ^1^H ^15^N HSQC spectra to pH induced chemical shift variations. The chemical shift values were found to be quite consistent for such comparisons as long as an internal standard (such as derivatized maleic acid in this work) was used for referencing.

**Figure 6:**
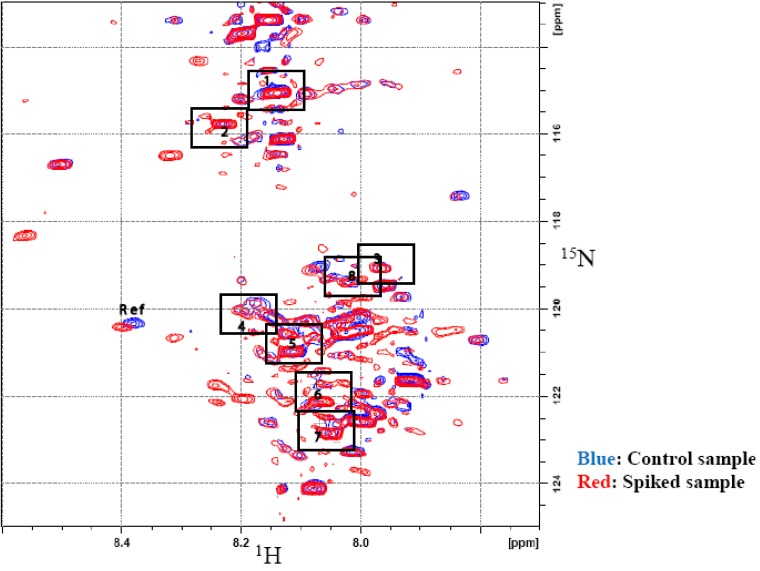
2D ^1^H-^15^N HSQC spectra of control urine (Blue) and urine spiked with several metabolites of interest (Red). Chemical shifts of the spiked compounds are indicated with boxes and are referenced to the metabolite information appearing in Table 1.

**Figure S2:**
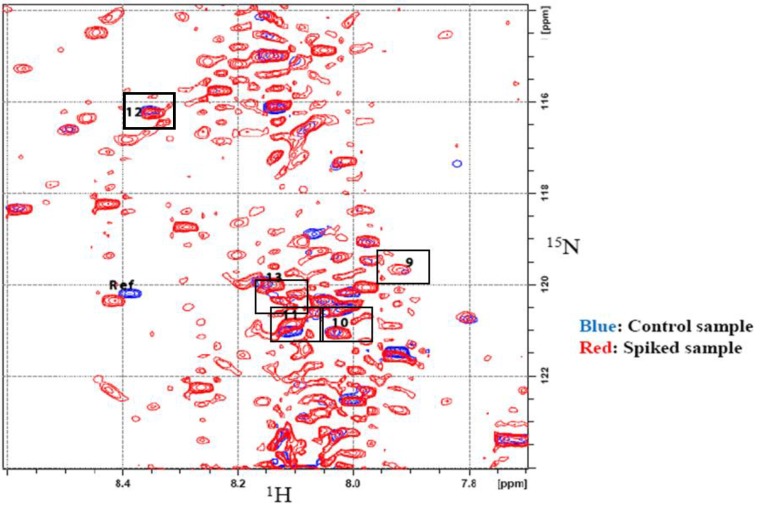
2D ^1^H-^15^N HSQC spectra of control urine (Blue) and urine spiked with several metabolites of interest (Red). Chemical shifts of the spiked compounds are indicated with boxes and are referenced to the metabolite information appearing in Table 1.

Spiking experiments were conducted to confirm the assignments. Spiking was performed using three pre-concentrated urine samples, of which two were spiked with aliquots from stock solutions of the tentatively assigned metabolites, while the third sample served as a control. Each control sample contained an equimolar amount of maleic acid, used as a referencing standard. All samples were then derivatized with ^15^N-ethanolamine and 2D HSQC experiments were then performed. The tentatively assigned metabolites were confirmed by first overlapping the HSQC spectra of the control and the spiked samples and then observing and measuring the change in intensities of the contours from the 2D spectra of the spiked samples and the control. Figure 6 shows the overlapped spectra of the control sample and urine spiked with compounds 1–8 (additional data for spiked compounds 9–13 are shown in Supplementary Figure S2). The list of metabolites and their assigned chemical shifts are summarized in Table 1.

**Figure S3:**
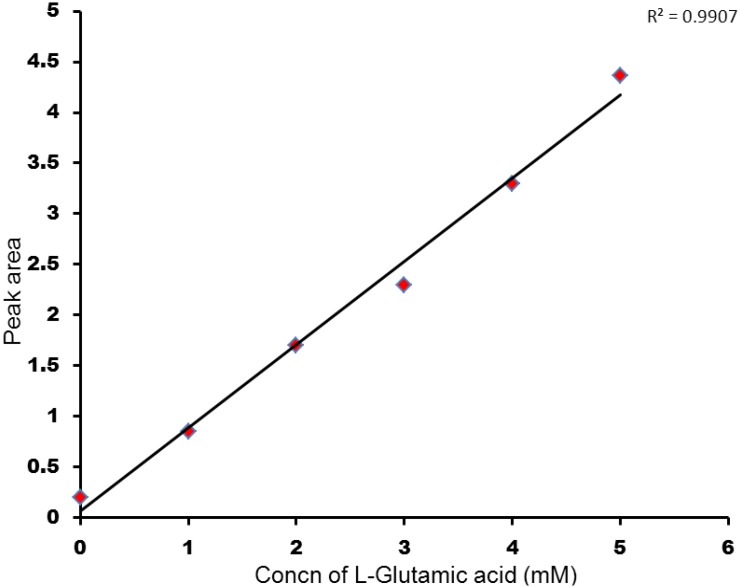
Calibration curve for varying concentrations of ^15^N isotope labeled L-glutamic acid spiked human urine.

We also investigated the quantitative abilities of our derivatization and LC-NMR method using glutamic acid in a bio-fluid matrix. L-glutamic acid, a key molecule in cellular metabolism, has signals that are difficult to detect without ambiguity in the ^1^H-NMR spectrum of human urine owing to extensive peak overlap and its low concentration. A calibration curve generated by standard addition of ^15^N isotope labeled glutamic acid into a sample of derivatized urine shows a good linear relationship between the concentration of derivatized L-glutamic acid and the NMR peak area with a coefficient of regression (R^2^) of ~0.99 (Figure S3). This implies that the isotopic tagging strategy could be implemented in order to estimate the amounts of endogenous metabolites present in any given urine sample due to two major factors; namely the accuracy of assignments of the NMR chemical shift values and their improved detection. The increased resolution afforded by the isotope labeling strategy, combined with the chromatographic resolution, is highly useful for distinguishing the metabolite signals and allowing good quantitation.

**Figure S4:**
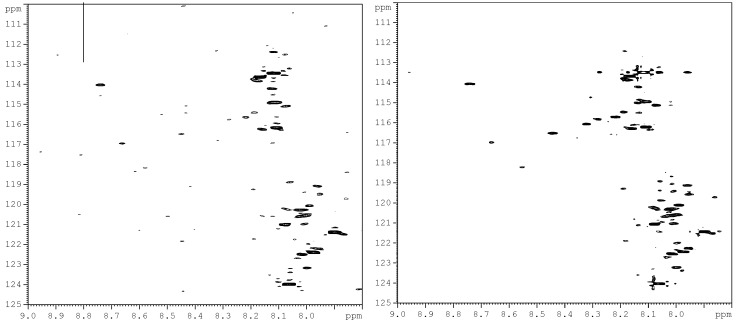
Typical 2D ^1^H-^15^N HSQC spectra of the same fractions as shown in Figure 3b, collected for two isotope tagged urine samples in the first dimension separation. Very similar spectral peak patterns were observed for the same fractions with minor differences arising from the altered metabolic content between the samples.

The chromatographic separation in the first dimension was useful in the elimination of several of the non-carboxyl containing components of urine and isolation of almost all the tagged components to a confined region in the chromatogram. The inability to resolve the tagged components in a single chromatographic step can be attributed mainly to the complexity of the sample matrix that contains many products of metabolism. In the second chromatographic separation, a sodium acetate buffer of pH 4.0 was used as the aqueous phase. This approach facilitated the resolution of the structurally similar metabolites, which eluted in order of their acid dissociation constants. Those metabolites with lower dissociation constants eluted later in the run owing to an increased retention resulting from their acquisition of a negative charge, and hence acted as extremely polar molecules. The reproducibility of the chromatographic runs was a prime factor towards obtaining reliable assignments. While multiple collections of fractions of interest were made, the NMR spectra of the same fractions from different cycles of collection showed good consistency in terms of quality and peak content (Figure S4).

1D ^1^H-NMR-based metabolomics typically and necessarily focuses on metabolites with relatively high concentrations (>0.1 mM). These metabolites often appear in multiple metabolic pathways and are often non-specific to different pathological and physiological roles. These issues consequently limit the ability to draw reliable conclusions from the analytical data. Alternatively, signals from lower concentration metabolites, which may have improved potential for use as biomarkers, are often buried beneath the dominant NMR signals of higher concentration metabolites or solvent signals. The use of chromatographic separation in conjunction with the ^15^N isotope tagging procedure can potentially circumvent some of these limitations. The data in Table 1, comprised of unambiguous assignments for 13 metabolites in human urine, represent a significant extension of the library of currently assigned ^15^N tagged compounds [40]. Significant improvements in the limits of detection can be inferred from the assignment of β-alanine, which has typical levels in urine of healthy adults of the order of a few µM, thus, making its detection in bio-fluids rather difficult by standard NMR. Additionally, several of the metabolites in Table 1 have potential clinical applications, which represent a highly favorable development for the future of NMR based metabolite profiling. For example, a high level of phenyl acetic acid is a marker of end-stage renal disease [46], 4-hydroxyphenylacetatic acid is associated the metabolic disorder, tyrosinemia [38] and suberic acid is associated with fatty acid oxidation disorder [47]. In the future, we plan to investigate the use of this chromatographic approach in conjunction with micro-coil NMR probes [20,21,22,23], which can potentially offer the ability to improve signal to noise levels for metabolites in volume limited samples, or to reduce the LOD after sample concentration.

## 4. Conclusions

The application of a two dimensional HILIC chromatography approach to resolve and identify several ^15^N-ethanolamine derivatized carboxyl containing metabolites in human urine in a reproducible fashion with good sensitivity has been demonstrated. This approach offers good promise for NMR based metabolite profiling applications, in which there is a major requirement for unambiguous assignment of signals arising from lower concentration species. Care should, however, be exercised, particularly when identifying peaks in the overlapped spectral regions; in such cases, other means such as detection by mass spectrometry or additional conventional NMR experiments should be used in addition to spiking to fully confirm metabolite identity.
